# Water inside β-cyclodextrin cavity: amount, stability and mechanism of binding

**DOI:** 10.3762/bjoc.15.163

**Published:** 2019-07-17

**Authors:** Stiliyana Pereva, Valya Nikolova, Silvia Angelova, Tony Spassov, Todor Dudev

**Affiliations:** 1 Faculty of Chemistry and Pharmacy, Sofia University “St. Kl. Ohridski”, 1164 Sofia, Bulgaria; 2 Institute of Organic Chemistry with Centre of Phytochemistry, Bulgarian Academy of Sciences, 1113 Sofia, Bulgaria

**Keywords:** β-cyclodextrin, DFT calculations, DSC/TG experiments, hydration, macrocycles, thermodynamic characteristics

## Abstract

Cyclodextrins (CDs) are native host systems with inherent ability to form inclusion complexes with various molecular entities, mostly hydrophobic substances. Host cyclodextrins are accommodative to water molecules as well and contain water in the native state. For β-cyclodextrin (β-CD), there is no consensus regarding the number of bound water molecules and the location of their coordination. A number of intriguing questions remain: (1) Which localities of the host’s macrocycle are the strongest attractors for the guest water molecules? (2) What are the stabilizing factors for the water clusters in the interior of β-CD and what type of interactions between water molecules and cavity walls or between the water molecules themselves are dominating the energetics of the β-CD hydration? (3) What is the maximum number of water molecules inside the cavity of β-CD? (4) How do the thermodynamic characteristics of β-CD hydration compare with those of its smaller α-cyclodextrin (α-CD) counterpart? In this study, we address these questions by employing a combination of experimental (DSC/TG) and theoretical (DFT) approaches.

## Introduction

Cyclodextrins (CDs), a family of enzymatically modified starches, are widely used as host macrocycles in forming inclusion complexes with various molecular entities of interest to food industry, pharmacology, cosmetics, catalysis, and environmental protection [1–8]. The CD exterior is hydrophilic due to the two rims (upper and lower rim) decorated with OH groups, whereas the interior cavity is relatively hydrophobic. As a result, these hosts can accommodate mostly hydrophobic substances inside their cavities. Note, however, that cyclodextrins are quite accommodative to water molecules as well: at ambient conditions, the free cyclodextrins represent undefined hydrates where the entrapped water has been suggested to stabilize the crystal lattice [1]. Upon binding, the incoming guest molecule competes with the cavity-bound water and can displace (completely or partially) the hydration content of the host molecule [2]. Most of these complexation reactions take place in aqueous solutions, thus it is important to understand the mechanism and energetics of interactions between the water molecules and the components of the supramolecular host–guest structures.

The water content of CDs has been a subject of numerous investigations. The data (both experimental and theoretical) are scattered and, still, no consensus has been reached on the number and position of water molecules, and the energetics of the hydration/dehydration of cyclodextrins. The results conflict mostly on the number of bound water molecules and the location of their coordination (inside/outside the host cavity). For β-CD, the experimentally established water content varies between 9 and 12 molecules: Sabadini et al. [9] have found 9.6 H_2_O/β-CD, Specogna 10 H_2_O [10], Bilal [11] and Nakai [12] between 10 and 12 H_2_O, Seidel [13] 10.41 H_2_O, Pereva [14] 10 H_2_O and Betzel, Lindner and Saenger between 9.4 and 12 H_2_O [15–17]. The authors of the latter study (Saenger) have concluded that the water molecules located inside and outside the cavity (in the interstices) are disordered and mobile, and that the OH groups of the host β-CD may rotate [17]. Studies with molecular dynamics simulations have found only four water molecules inside the host β-CD cavity [18].

The energetics of the CD hydration/dehydration have been investigated as well. Experimental studies have been able to differentiate between the strongly and weakly connected water molecules to the CDs [5,10–11,14]. Furthermore, TG/TGA and DSC experiments have demonstrated that the water content of β-CD is released in one-step at temperatures between 35–120 °C accompanied by a large endothermic effect [11]. The enthalpy of this process has been found to be ≈40–45 kJ mol^−1^ [10–11,14].

The brief literature survey outlined above shows that, although a significant body of information has been accumulated on the β-CD hydration/dehydration, the intimate mechanism of the process is still not completely understood leaving several outstanding questions unanswered: 1) Which localities of the host’s macrocycle are the strongest attractors for the incoming water molecules? (2) What are the major factors contributing to the stability of the water clusters entrapped in the β-CD interior and what type of interactions between water molecules and cavity walls or between the water molecules themselves are dominating the energetics of the β-CD hydration? 3) What is the maximum number of water molecules inside the cavity of β-CD? 4) How do the thermodynamic characteristics of β-CD hydration compare with those of its smaller α-CD counterpart?

In this study, we endeavor to address these questions by employing a combination of experimental (DSC/TG) and theoretical approaches (DFT). Our findings illuminate the mechanism of β-CD hydration at an atomic level and disclose the major factors controlling this process. A similar combined computational and experimental study of α-CD instead of β-CD hydration/dehydration has already been performed by us [19].

## Results and Discussion

### Nonhydrated β-CD

**Figure 1:**
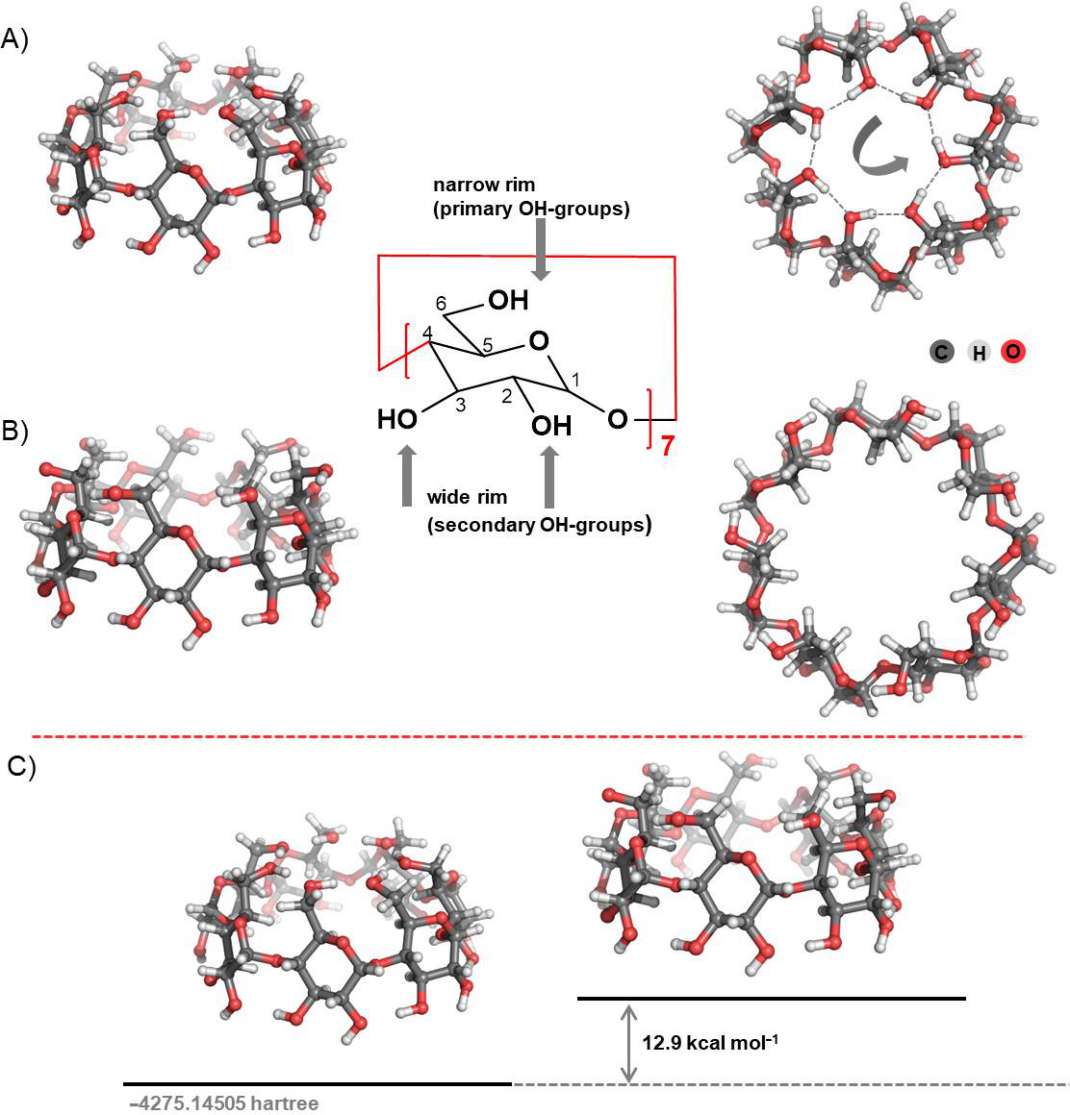
M062X/6-31G(d,p) optimized structure of nonhydrated β-CD in two projections (left: a side view and right: a top view): (A) structure with oppositely oriented intramolecular hydrogen bonds at both rims: looking from the top (the narrow rim side) the direction of the wide rim hydrogen bonds is CW, while the orientation of the narrow rim hydrogen bonds is CCW; (B) structure with “open” narrow rim; and (C) energy difference between these two configurations at the M062X/6-311++G(d,p)//M062X/6-31G(d,p) level of theory.

The nonhydrated β-CD molecules possess a nearly perfect 7-fold symmetry. The primary hydroxy groups (positioned at the upper/narrow belt) can be arranged in two alternative ways: (i) facing inward and creating a hydrogen-bond girdle (“head–tail” arrangement) that greatly decreases the size of the cavity vestibule (Figure 1A), and (ii) facing outward thus widening the aperture (“open” configuration, Figure 1B). Notably, in the “open” configuration the primary hydroxy groups do not participate in intramolecular hydrogen bond interactions. Relatively weak hydrogen bonds are formed between the secondary hydroxy groups in the wider/lower rim of the molecule. The calculations reveal that the “head–tail” arrangement of the narrow belt is energetically favoured over the “open” configuration (by 12.9 kcal mol^−1^; Figure 1C) thus the “head–tail” structure was employed in the subsequent evaluations.

### Hydrated β-CD

#### Water binding to the central cavity

**Figure 2:**
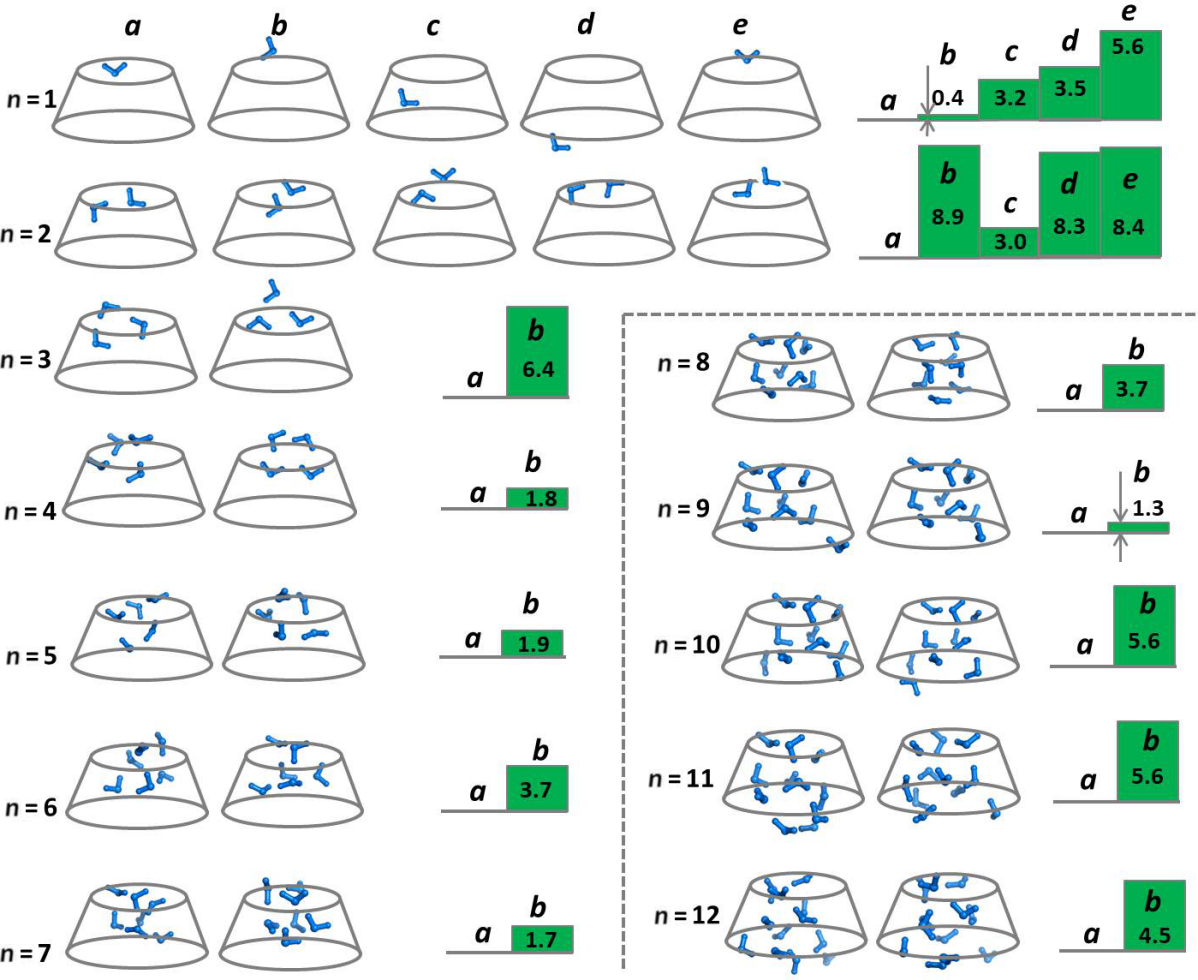
Schematic representation of β-CD–*n*H_2_O complexes (where *n* = 1–12) with water molecules/clusters located at different positions, and M062X/6-311++G(d,p)//M062X/6-31G(d,p) calculated relative enthalpies of the respective complexes.

β-CD hydrates containing one to twelve water molecules bound at various localities in the host molecule interior were modelled and energetically optimized (Figure 2). The hydration mechanism involving sequential binding of individual water molecules to α-CD [19] had been found to be the preferred fashion of coordination (as opposed to the bulk hydration by pre-formed water clusters). Thus only this mode of hydration was considered for β-CD. The cavity of the host’s macrocycle was probed for places/spots exhibiting enhanced affinity for the incoming water molecules. The first entrapped water molecule can bind to either rim (*n* = 1; Figure 2, structures **a**, **b**, **d**, **e**) or the cavity walls (*n* = 1; Figure 2, structure **c**). As the calculated relative enthalpies of the resulting complexes (Figure 2) suggest, the structures with a water molecule positioned at the narrow rim of β-CD (*n* = 1; Figure 2, structures **a** and **b**) appeared to be the most stable ones. Therefore, the upper rim with the “head–tail” assembly of the primary hydroxy groups can be considered as a major attractor (“hot” spot) for the incoming water. Similar results had been obtained for the positioning of the first water molecule hosted by the smaller α-CD counterpart [19].

**Figure 3:**
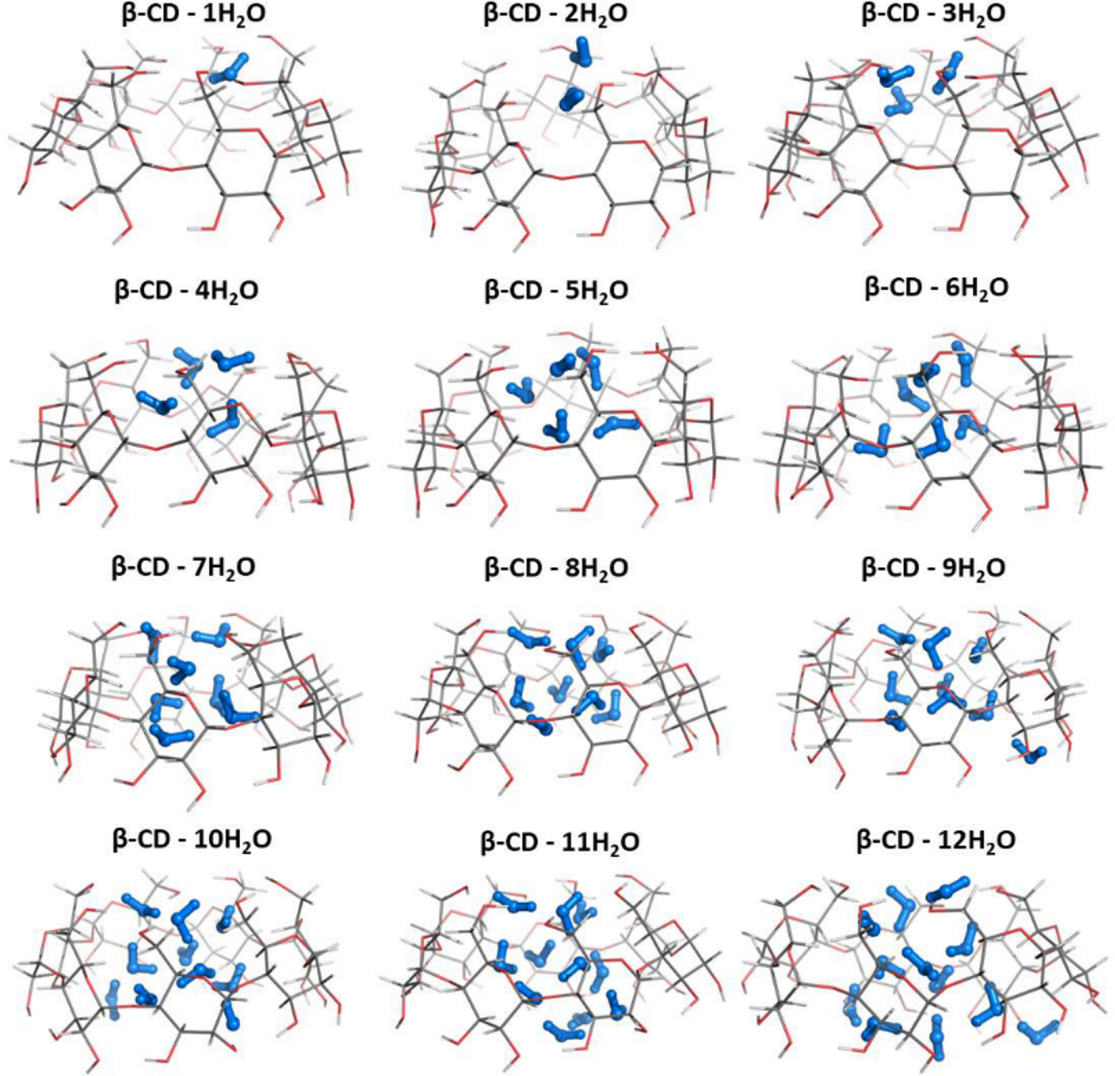
M062X/6-31G(d,p) optimized structures of the most stable (**a** structures from Figure 2) β-CD–*n*H_2_O complexes (*n* = 1–12).

**Figure 4:**
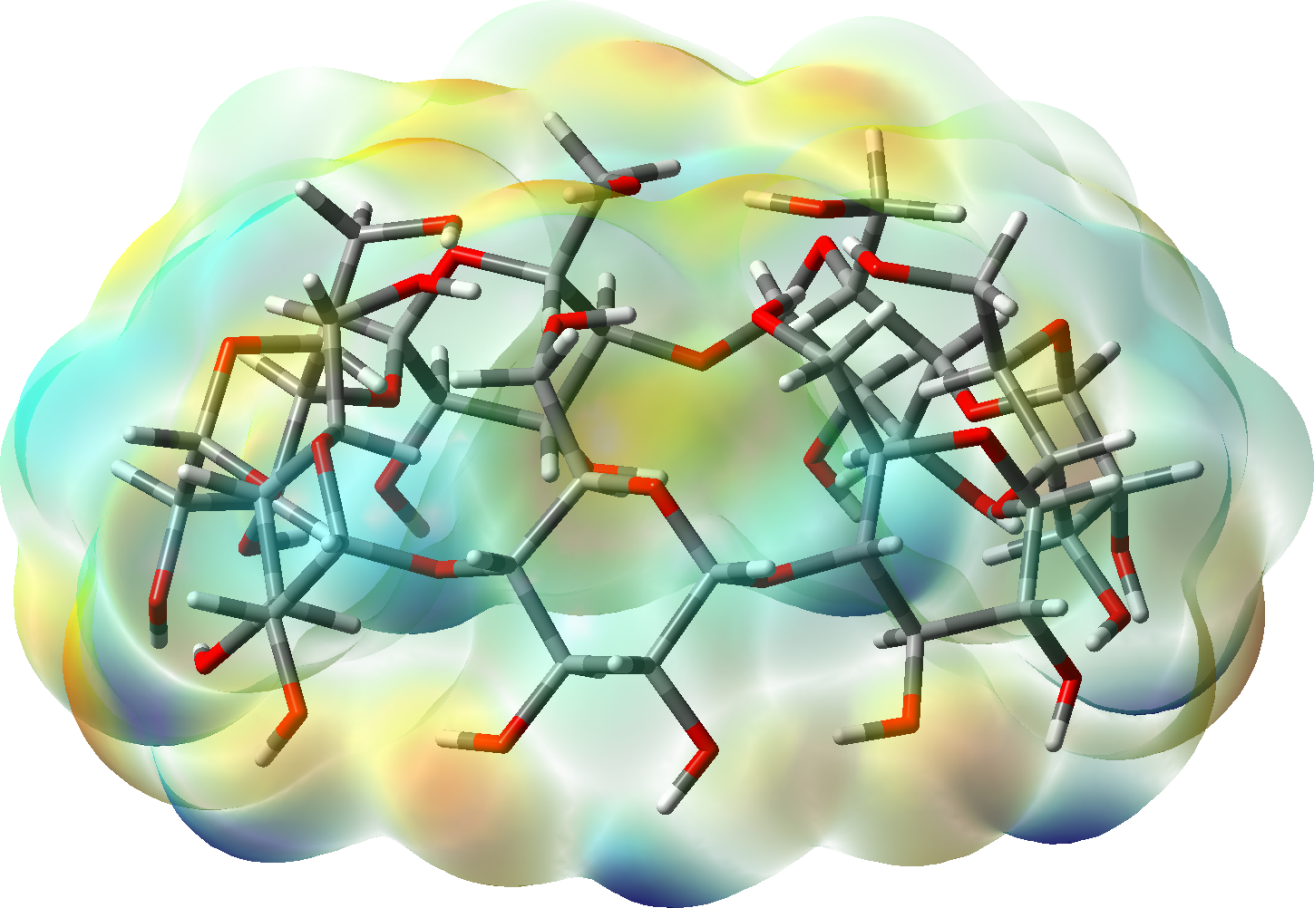
Graphical model of the β-CD electron density (isovalue = 0.002), mapped with electrostatic potential (color scheme used: blue for the positive surface map values and red/yellow for negative ones).

In constructing the heavier β-CD hydrates containing greater numbers of water molecules, *n* > 1, each subsequent water molecule was appropriately inserted to the β-CD−(*n*−1)H_2_O complex so that it would maximize its interactions with the neighbouring H_2_O molecules or hydroxy groups from the upper/lower belts. Our calculations suggest that for each hydrate the cluster of hydrogen-bonded water molecules involving the water ligand already bound to the upper belt is the energetically favoured structure (**a** structures for *n* = 2–12 in Figure 2) over the other alternative constructs. For the β-CD–2H_2_O complex, binding of two water molecules to the narrow rim is the preferred mode of coordination (*n* = 2; Figure 2, structure **a**). In this construct the water dimer not only connects two oppositely located OH groups from the rim, but additionally interacts with four other OH groups and forms an elaborate hydrogen-bond network that almost occludes the aperture of the rim. Furthermore, the results obtained demonstrate that the β-CD–3H_2_O complexes with all the three water molecules bound to the narrow rim (the third one located below the plane of the narrow rim and hydrogen bonded to another water and OH residue from the rim) is more stable (*n* = 3; Figure 2a), than the respective complex with the third water molecule located outside the cavity (Figure 2b). For larger water clusters (*n* > 3) filling up the cavity continues gradually from the narrow to the wide rim as the additional water molecule engages in hydrogen bond interactions predominantly with fellow waters from the (*n −* 1) clusters (Figure 2 and Figure 3). It can be concluded (similarly to the previously examined α-CD hydration [19]) that the first water molecule coordinated to the narrow rim OH groups plays a pivotal role in the formation and stabilization of the β-CD polyhydrates acting as an anchor for the subsequently formed hydrogen-bonded water clusters inside the host cavity. The attraction and crowding of water molecules at the “hot spot” narrow belt is not unexpected in view of the higher electron density concentrated in this pre-organized by hydrogen bonding location (Figure 4).

Table 1 lists the enthalpies evaluated at the M062X/6-31G(d,p) level of theory for the stepwise water coordination to the host macrocycle in both the gas phase and aqueous solution. Negative Δ*H*^1^ and Δ*H*^78^ values imply favorable interactions in both media. Similar trends were observed for enthalpies evaluated at the M062X/6-311++G(d,p)//M062X/6-31G(d,p) level of theory (Supporting Information File 1, Table S1). As a general trend, it was observed that expanding the intramolecular hydrogen bond network upon each H_2_O addition (comprising the water–water and water–cyclodextrin interactions) enhances the efficiency of the hydration process. For example, the β-CD dihydrate (Figure 3) containing two water molecules bridging the oppositely placed hydroxy groups from the upper belt thus increasing the number of hydrogen bonds, is characterized with a lower (i.e., more favorable) enthalpy of formation than other structures possessing less elaborate hydrogen bond networks (compare Δ*H*^1^/Δ*H*^78^ for reaction 2 with those of reactions 1 and 3 in Table 1). Generally, increasing the polarity of the medium (higher dielectric constant) attenuates the energies of complex formation although the enthalpies remain negative. Adding an 11th water molecule to the water cluster appears to trigger significant structural changes of the host β-CD structure, which starts to distort in order to accommodate the increased number of entrapped water molecules (Figure 3). Thus, our theoretical results (which are in line with the experimental observations, see below) imply that 10 water molecules entrapped inside the cavity is the upper limit of the (non-distorted) β-CD hydration.

**Table 1:** Gas phase enthalpies (Δ*H*^1^) calculated at the M062X/6-31G(d,p) level of theory and enthalpies in water environment (Δ*H*^78^) (in kcal mol^−1^) for the most stable β-CD–*n*H_2_O (*n* = 1–12) complex formation.

	Δ*H*^1^	Δ*H*^78^

1. β-CD + H_2_O → β-CD–H_2_O	−7.6	−2.8
2. β-CD–H_2_O + H_2_O → β-CD–2H_2_O	−18.1	−12.2
3. β-CD–2H_2_O + H_2_O → β-CD–3H_2_O	−14.3	−8.1
4. β-CD–3H_2_O + H_2_O → β-CD–4H_2_O	−10.1	−6.1
5. β-CD–4H_2_O + H_2_O → β-CD–5H_2_O	−17.9	−10.0
6. β-CD–5H_2_O + H_2_O → β-CD–6H_2_O	−18.6	−8.8
7. β-CD–6H_2_O + H_2_O → β-CD–7H_2_O	−17.1	−12.0
8. β-CD–7H_2_O + H_2_O → β-CD–8H_2_O	−10.6	−5.0
9. β-CD–8H_2_O + H_2_O → β-CD–9H_2_O	−10.8	−3.1
10. β-CD–9H_2_O + H_2_O → β-CD–10H_2_O	−10.1	−5.5
11. β-CD–10H_2_O + H_2_O → β-CD–11H_2_O	−21.8	−14.5
12. β-CD–11H_2_O + H_2_O → β-CD–12H_2_O	−8.9	−1.8

Cumulative formation energies calculated at the M062X/6-31G(d,p) level of theory of β-CD–aqua complexes with 1 to 10 bound water molecules in both the gas phase and water environment are given in Table 2. Similar trends were observed for energies evaluated at the M062X/6-311++G(d,p)//M062X/6-31G(d,p) levels of theory (Supporting Information File 1, Table S2). The results obtained suggest that the process is energetically favorable in both dielectric media. As compared to the respective α-CD hydration mechanism [19], the values are more negative (higher in absolute value) implying less hydrophobicity of the β-CD cavity (probably related to the larger internal volume) relative to that of its α-CD counterpart.

**Table 2:** Formation energies (in kcal mol^−1^) calculated at the M062X/6-31G(d,p) level of theory of β-CD–*n*H_2_O (*n* = 1–10) complexes with nonhydrogen-bonded water molecules.

	Δ*H*^1^	Δ*H*^78^

β-CD + 2H_2_O → βCD–2H_2_O	−25.7	−15.0
β-CD + 3H_2_O → β-CD–3H_2_O	−40.0	−23.1
β-CD + 4H_2_O → β-CD–4H_2_O	−50.1	−29.2
β-CD + 5H_2_O → β-CD–5H_2_O	−68.0	−39.2
β-CD + 6H_2_O → β-CD–6H_2_O	−86.6	−48.0
β-CD + 7H_2_O → β-CD–7H_2_O	−103.8	−60.1
β-CD + 8H_2_O → β-CD–8H_2_O	−114.3	−65.0
β-CD + 9H_2_O → β-CD–9H_2_O	−125.1	−68.1
β-CD + 10H_2_O → β-CD–10H_2_O	−135.2	−73.6

#### Thermal dehydration of β-CD

**Figure 5:**
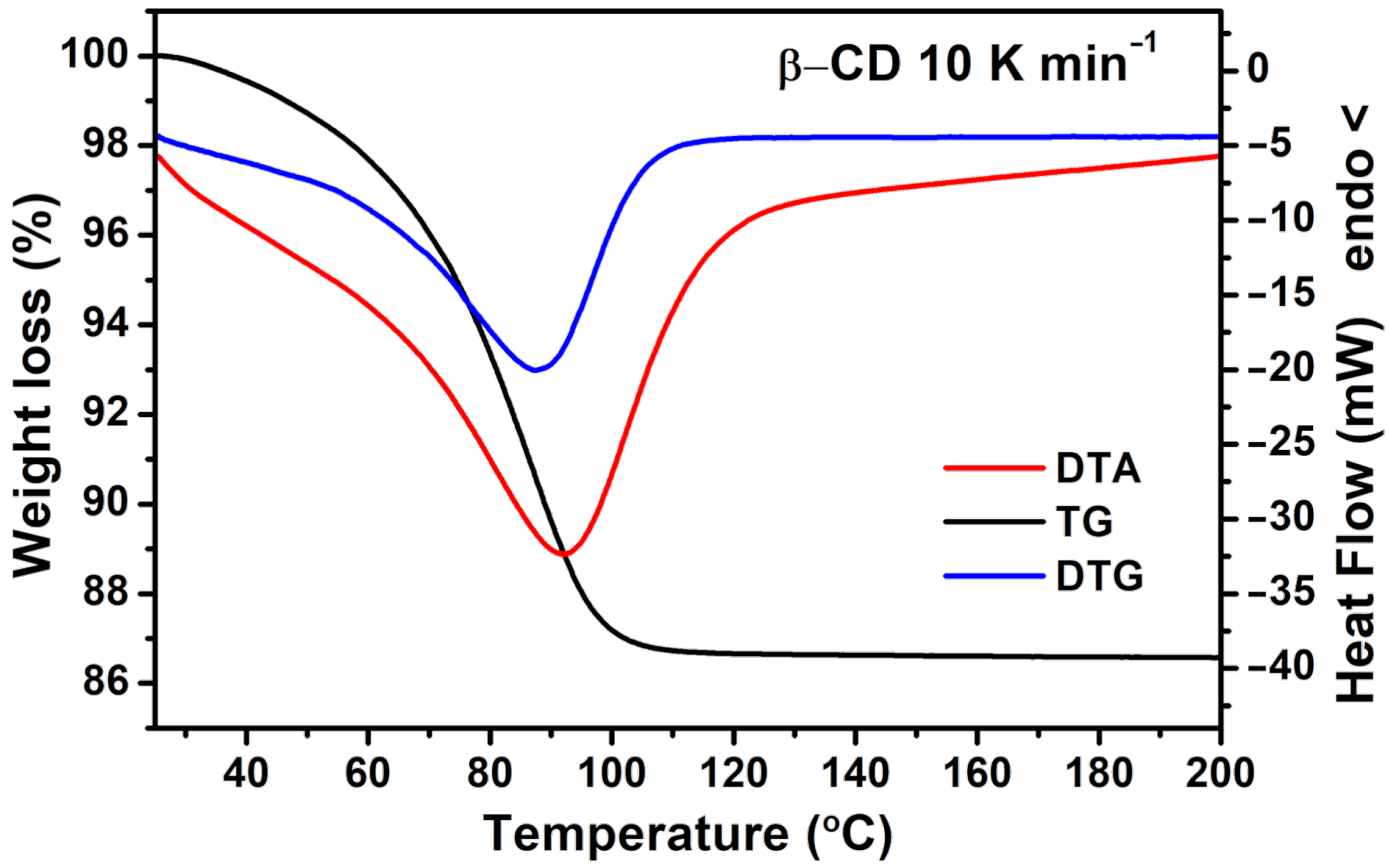
Thermal behavior of β-CD.

The thermal behavior of β-CD in the temperature range from 30 °C to 200 °C, in which only its dehydration takes place, is presented in Figure 5.

**Figure 6:**
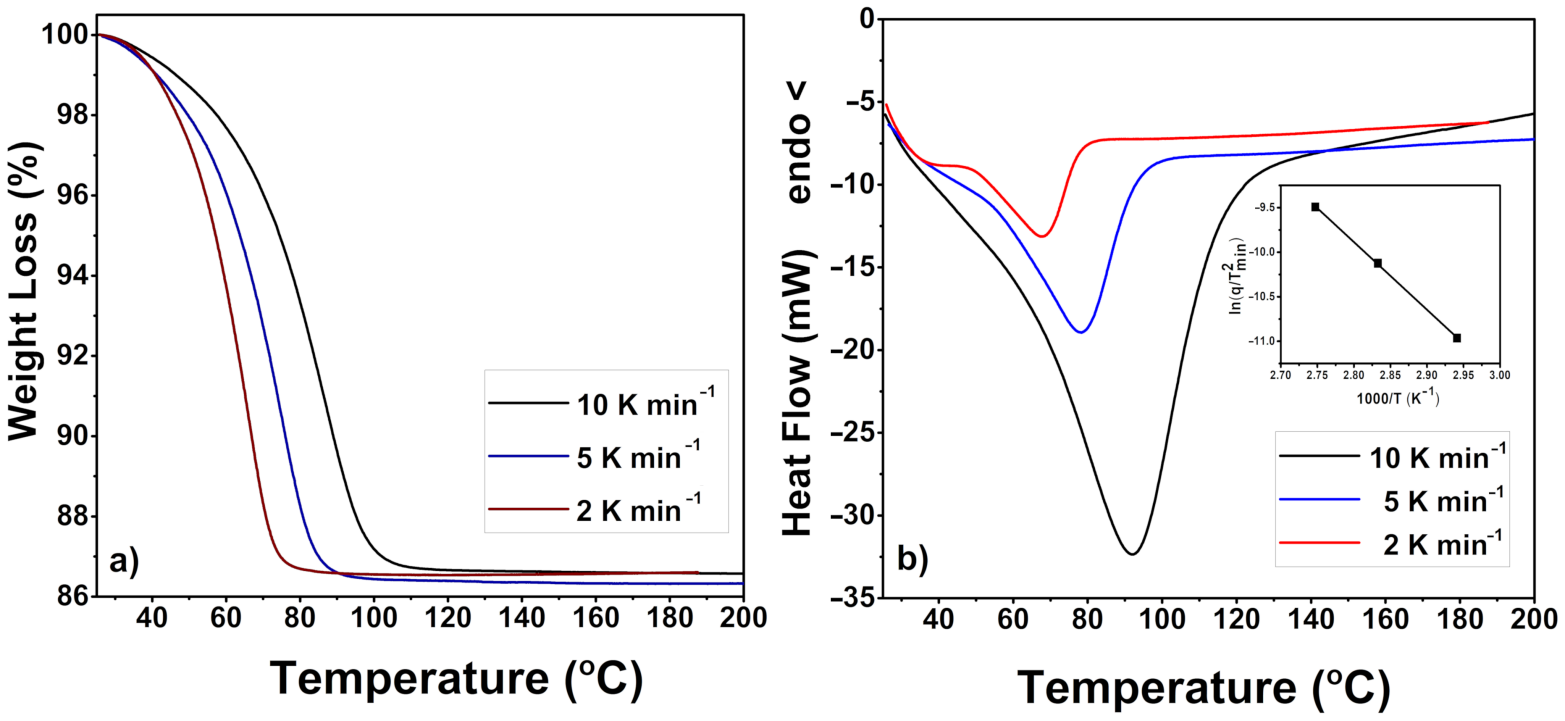
Thermal behavior of β-CD: TG curves (a) and DTA scans (b).

Both DTA and TG/DTG curves reveal one step dehydration reactions in the range 30–100 °C. This result differs from the observed stepwise weight reduction (due to water release) for α-CD [19]. Because the only volatile component in this temperature range is water, its amount was determined to be 10 water molecules liberated per β-CD molecule. Similar results were shown by other authors as well [11,13], although there are some differences in the reported data mainly due to the different conditions in which β-CD was stored. It has to be mentioned that when β-CD, used in our study, is stored at ambient conditions and the dehydration is measured over different periods during 1 year, the difference in the amount of included water is negligibly small (Figure 6а). Furthermore, the dehydration was carried out at different heating rates to check if it is a one-step process.

The observed one-step dehydration (or two highly overlapping dehydration reactions) implies that, unlike α-CD, the water in β-CD is tightly connected in a cluster, which in turn is easily detached from the much more flexible (compared to α-CD) β-CD molecule. Only when measured at lower heating rates the dehydration thermograms reveal a small low-temperature endothermic peak overlapped with the main dehydration peak (Figure 6b). This might be associated with some differences in the binding energies of the water molecules inside β-CD.

Both DTA and DSC analyses of β-CD allow to determine with sufficient precision the enthalpy of β-CD dehydration. The value of 35–40 kJ mol^−1^ H_2_O (8.4–9.6 kcal mol^−1^ H_2_O) is close to that found by Bilal et al. [11] and a little higher than that experimentally obtained for α-CD (5.3 ± 0.9 and 7.2 ± 0.6 kcal mol^−1^ H_2_O for the first and second water release, respectively) [19]. The variation of the endothermic peak maximum with the rate of heating in DTA permits an activation energy of β-CD dehydration about 60 kJ mol^−1^ (14.3 kcal mol^−1^) to be also estimated, applying the Kissinger method [20] (Figure 6b inset). This value is similar to those available in the literature for β-CD dehydration [21].

## Conclusion

The predominantly hydrophobic β-CD cavity, similarly to that of its smaller α-CD counterpart, can accommodate polar substances and water molecules in particular. The process of water inclusion into β-CD is energetically favorable. Both experiments and theory agree that up to ten water molecules can be accommodated internally by the β-CD host. The first incoming water molecules cluster around the narrow belt due to the higher electron density concentrated in this location. Inside the β-CD cavern a water cluster is formed by a stepwise consecutive coordination of water guests. The cluster is stabilized by a network of hydrogen bonds mostly between the water molecules themselves and interactions between water ligands and cyclodextrin walls. The general trends, observed earlier for α-CD hydration, hold for β-CD as well: the hot spot of the host molecule with the highest affinity for water molecule(s) is the narrow rim. Interestingly, the α-CD–H_2_O and β-CD–H_2_O complex formation is characterized by very close Δ*H*^78^ values (−2.6 and −2.8 kcal mol^−1^, respectively). Note, however, that a substantial difference between the α-CD and β-CD dehydration exists: while the α-CD dehydration follows a stepwise individual water release [19], the β-CD dehydration is characterized with a one-step bulk release of the water content (with enthalpy of 8.4−9.6 kcal mol^−1^). This strongly suggests that the hydration of β-CD is dominated by water–water hydrogen bond interactions rather than water–host cavity interactions. In contrast, the shared interactions between water molecules and cavity walls to the hydration process is more significant in the smaller α-CD counterpart favoring a consecutive water release upon heating.

## Experimental

### Materials

β-CD was bought from Wacker Chemie AG (CAVAMAX W7 FOOD) with purity of ≥98% and was used for the thermal analyses without any further purification.

### Experimental measurements

The thermal behavior of β-CD was studied by a Perkin-Elmer differential thermal analysis with thermogravimetry (DTA/TG) and by differential scanning calorimetry (DSC) using a Perkin-Elmer DSC-7. The samples were heated from room temperature (25 °C) to 200 °C with different constant scanning rates – from 2 to 10 K min^−1^ in pure nitrogen atmosphere. Temperature and heat flow calibration of the DSC was made by evaluating the melting peak of pure In and Zn. Dry nitrogen was used as purge gas at a fixed flow rate of 20 mL min^−1^. All DSC and DTA/TG measurements were repeated three times to ensure reproducibility and accuracy of the determined quantities.

### Computational details

Different molecular modeling methods (quantum mechanics (QM), molecular dynamics (MD), docking and quantitative structure activity relationships (QSARs)) can be applied in studying the structure, dynamics, and energetics of the host CD systems. However, the results from different modeling (or experimental) approaches are sometimes conflicting and contradictory. There is no “standard” method to investigate the complexation behavior of CDs. Thus, in our study a computational quantum mechanical modelling method, namely density functional theory (DFT) was chosen to investigate the electronic structure and properties of the objects. The geometries of β-CD, water molecule/clusters and hydrated complexes were optimized at the M062X/6-31G(d,p) level of theory and the electronic energy, *E*_el_, of each structure was evaluated using the Gaussian 09 program [22]. This method/basis set combination was chosen as it reliably reproduced the geometry of model systems of similar structure/composition [23]. In the study of the hydration of α-CD it was tested with respect to the experimentally determined structure of α-CD and demonstrated to be appropriate for structural modeling of cyclodextrins. A computational protocol similar to that employed in our previous work on α-CD hydration was used [19]. We performed single point calculations (using the optimized M062X/6-31G(d,p) structures) and evaluated *E*_el_ at the M062X/6-311++G(d,p) level of theory in order to assess the effect of the increased sophistication of the employed basis set on the calculated energies. Electronic energies obtained at both levels of theory (M062X/6-31G(d,p) and M062X/6-311++G(d,p)//M062X/6-31G(d,p)) were used alongside in the subsequent evaluations. The calculations at the two theoretical levels were previously validated against experimental data (dissociation energy, D_0_, in the gas phase) on water dimer. Frequency calculations for each M062X/6-31G(d,p) optimized construct were performed at the same level of theory. No imaginary frequency was found for the lowest energy configurations of any of the optimized structures. The frequencies were used to compute the respective thermal energy correction, *E*_th_, including zero point energy, to the electronic energy yielding the gas phase enthalpy of the molecule/complex at *T* = 298.15 K (Equation 1).

[1] $$H^{1}=E_{el}+E_{th}$$

Solvation energies were evaluated by employing the solvation model based on density (SMD) scheme as encoded in the Gaussian 09 program package. Single-point calculations in water (with dielectric constant ε ≈ 78) at both levels of theory M062X/6-31G(d,p)//M062X/6-31G(d,p) and M062X/6-311++G(d,p)//M062X/6-31G(d,p) were performed on each fully optimized gas-phase structure. The difference between the gas phase and SMD energies was used to evaluate the solvation energy, ∆*E*_solv_^ε^, of the respective molecule/complex: ∆*E*_solv_^ε^ ≈ E_el_^ε^ − E_el_. Following the standard thermodynamic cycle approach, the solvation energies of the reactants and products were employed to calculate the enthalpy of complex formation, ∆*H*^ε^, in condensed medium (water, Equation 2):

[2] $$\Delta H^{\varepsilon }=\Delta H^{1}+\Delta E_{\text{solv}}{}^{\varepsilon }(\text{products})-\Delta E_{\text{solv}}{}^{\varepsilon }(\text{reactants)}$$

Basis set superposition errors (BSSE) were considered by following the counterpoise approach of Boys and Bernardi as implemented in Gaussian 09 package. The PyMOL software was used for creating the molecular graphics images [24].

## Supporting Information

File 1 Additional data from DFT computations at the M062X/6-311++G(d,p)//M062X/6-31G(d,p) level of theory.
