# Supplementary material for: Water inside β-cyclodextrin cavity: amount, stability and mechanism of binding
Source: Beilstein J Org Chem. 2019 Jul 17;15:1592–600. doi: 10.3762/bjoc.15.163 (PMC6664416; doi:10.3762/bjoc.15.163)
Supplement: File 1 — Additional data from DFT computations at the M062X/6-311++G(d,p)//M062X/6-31G(d,p) level of theory. [file Beilstein_J_Org_Chem-15-1592-s001.pdf]

BEILSTEIN JOURNAL OF ORGANIC CHEMISTRY

Supporting Information

for

Water inside β-cyclodextrin cavity: amount, stability and mechanism of binding

Stiliyana Pereva, Valya Nikolova, Silvia Angelova, Tony Spassov and Todor Dudev

Beilstein J. Org. Chem. **2019,** *15,* 1592–1600. doi:10.3762/bjoc.15.163

Additional data from DFT computations at the M062X/6-311++G(d,p)//M062X/6-31G(d,p) level of theory

License and Terms: This is a supporting information file under the terms of the Creative Commons Attribution License (http://creativecommons.org/licenses/by/4.0). Please note that the reuse, redistribution and reproduction in particular requires that the authors and source are credited.
The license is subject to the *Beilstein Journal of Organic Chemistry* terms and conditions: (https://www.beilstein-journals.org/bjoc)

Table S1: Enthalpies calculated at the M062X/6-311++G(d,p)//M062X/6-31G(d,p) level of theory in the gas phase (ΔH^1) and water environment (ΔH^{78}) (in kcal mol^{-1}) for the most stable β-CD–$n$$H_2O$ complex formation (n = 1–12).

	ΔH^1	ΔH^{78}
1. β-CD + H_2O → β-CD − H_2O	−2.7	1.9
2. β-CD − H_2O + H_2O → β-CD − 2H_2O	−15.7	−9.1
3. β-CD − 2H_2O + H_2O → β-CD − 3H_2O	−7.2	−0.3
4. β-CD − 3H_2O + H_2O → β-CD − 4H_2O	−7.0	−2.8
5. β-CD − 4H_2O + H_2O → β-CD − 5H_2O	−13.3	−5.1
6. β-CD − 5H_2O + H_2O → β-CD − 6H_2O	−13.8	−3.6
7. β-CD − 6H_2O + H_2O → β-CD − 7H_2O	−11.8	−6.6
8. β-CD − 7H_2O + H_2O → β-CD − 8H_2O	−4.7	2.3
9. β-CD − 8H_2O + H_2O → β-CD − 9H_2O	−6.1	0.3
10. β-CD − 9H_2O + H_2O → β-CD − 10H_2O	−0.2	0.9
11. β-CD − 10H_2O + H_2O → β-CD − 11H_2O	−17.0	−9.2
12. β-CD − 11H_2O + H_2O → β-CD − 12H_2O	−6.4	3.4

Table S2: Formation energies calculated at the M062X/6-311++G(d,p)//M062X/6-31G(d,p) level of theory (in kcal mol^{-1}) of β-CD–$n H_2O$ (n = 1−10) complexes with individual (nonhydrogen)-bonded water molecules.

	Δ*H*[1]	Δ*H*[78]
β-CD + $2H_2O$ → β-CD − $2H_2O$	−18.4	−7.1
β-CD + $3H_2O$ → β-CD − $3H_2O$	−25.6	−7.4
β-CD + $4H_2O$ → β-CD − $4H_2O$	−32.6	−10.2
β-CD + $5H_2O$ → β-CD − $5H_2O$	−45.9	−15.3
β-CD + $6H_2O$ → β-CD − $6H_2O$	−59.7	−18.9
β-CD + $7H_2O$ → β-CD − $7H_2O$	−71.5	−25.5
β-CD + $8H_2O$ → β-CD − $8H_2O$	−76.3	−23.3
β-CD + $9H_2O$ → β-CD − $9H_2O$	−82.3	−23.0
β-CD + $10H_2O$ → β-CD − $10H_2O$	−82.6	−22.1
